# Understanding and exploiting the roles of O-GlcNAc in neurodegenerative diseases

**DOI:** 10.1016/j.jbc.2023.105411

**Published:** 2023-10-31

**Authors:** Matthew R. Pratt, David J. Vocadlo

**Affiliations:** 1 Department of Chemistry and Department of Biological Sciences, University of Southern California, Los Angeles, California, USA; 2 Department of Chemistry, Simon Fraser University, Burnaby, British Columbia, Canada; 3 Department of Molecular Biology and Biochemistry, Simon Fraser University, Burnaby, British Columbia, Canada

**Keywords:** neurodegeneration, O-GlcNAc, synuclein, tau, protein synthesis, enzyme inhibitors, clinical development, Alzheimer's disease

## Abstract

O-GlcNAc is a common modification found on nuclear and cytoplasmic proteins. Determining the catalytic mechanism of the enzyme O-GlcNAcase (OGA), which removes O-GlcNAc from proteins, enabled the creation of potent and selective inhibitors of this regulatory enzyme. Such inhibitors have served as important tools in helping to uncover the cellular and organismal physiological roles of this modification. In addition, OGA inhibitors have been important for defining the augmentation of O-GlcNAc as a promising disease-modifying approach to combat several neurodegenerative diseases including both Alzheimer’s disease and Parkinson’s disease. These studies have led to development and optimization of OGA inhibitors for clinical application. These compounds have been shown to be well tolerated in early clinical studies and are steadily advancing into the clinic. Despite these advances, the mechanisms by which O-GlcNAc protects against these various types of neurodegeneration are a topic of continuing interest since improved insight may enable the creation of more targeted strategies to modulate O-GlcNAc for therapeutic benefit. Relevant pathways on which O-GlcNAc has been found to exert beneficial effects include autophagy, necroptosis, and processing of the amyloid precursor protein. More recently, the development and application of chemical methods enabling the synthesis of homogenous proteins have clarified the biochemical effects of O-GlcNAc on protein aggregation and uncovered new roles for O-GlcNAc in heat shock response. Here, we discuss the features of O-GlcNAc in neurodegenerative diseases, the application of inhibitors to identify the roles of this modification, and the biochemical effects of O-GlcNAc on proteins and pathways associated with neurodegeneration.

O-GlcNAc modifications (Fig. 1*A*) are an abundant intracellular form of glycosylation found in all metazoans, plants, and some microbes (1, 2, 3). In animals, a single GlcNAc monosaccharide is added onto serine residues or threonine residues by the enzyme O-GlcNAc transferase (OGT) and can be subsequently removed by another enzyme O-GlcNAcase (OGA) (4). The dynamics of individual O-GlcNAc sites vary, but this cycling mechanism enables O-GlcNAc to function as a regulator of protein biochemistry in response to various cellular stimuli. O-GlcNAc was first discovered by Torres and Hart in 1984 (5)through a series of enzymatic labeling experiments published in this journal. Specifically, they first metabolically labeled cells with {^3^H}-glucosamine, resulting in the endogenous production of radioactive, {^3^H}-GlcNAc-containing glycans. They then took advantage of a recombinant β1,4-galactoslytransferase (GalT) to label any terminal GlcNAc residues with {^14^C}-galactose. Surprisingly, they found that the majority of the resulting {^14^C}-Gal-β1,4-{^3^H}-GlcNAc was found within the interior of cells rather than on known cell surface glycoproteins. During the following ∼10 years, a series of experiments from several laboratories confirmed the presence of O-GlcNAc on a range of intracellular proteins and identified and cloned both OGT and OGA (6, 7, 8, 9, 10, 11), establishing a firm biological foundation for this critical post-translational modification.

**Figure 1:**
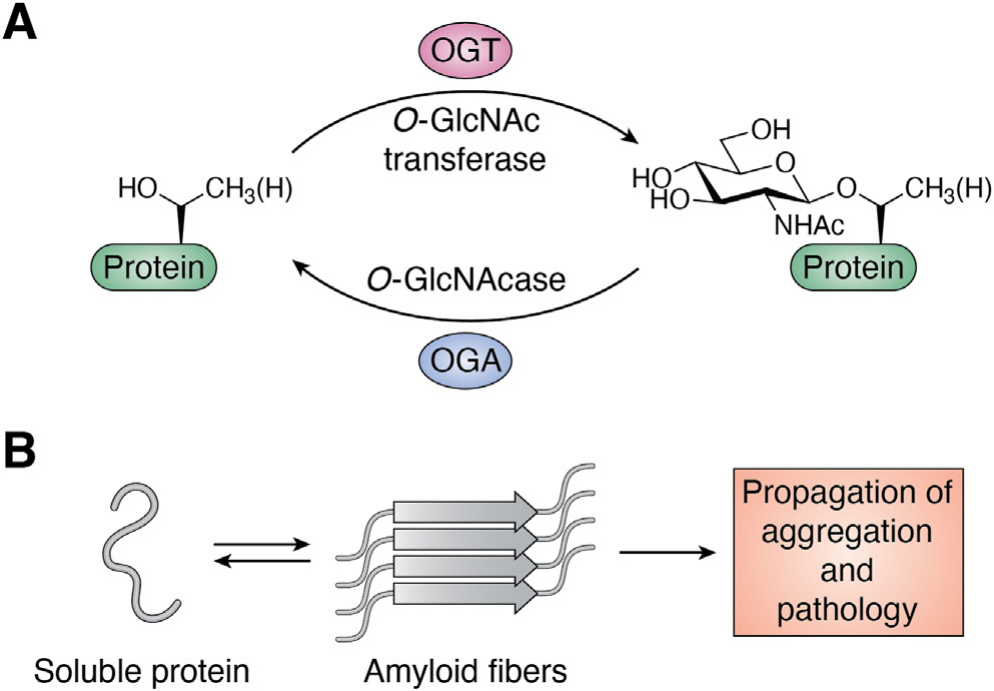
**O-GlcNAc and protein amyloid aggregation.***A,* O-GlcNAc is a dynamic monosaccharide modification of intracellular proteins mediated by the enzymes O-GlcNAc transferase (OGT) and O-GlcNAcase (OGA). *B*, neurodegenerative diseases are associated with the progressive formation of amyloid aggregates.

Since these initial field-establishing experiments, many laboratories have contributed to our understanding of O-GlcNAc in various areas of biology and human health, including neurodegenerative diseases (NDDs). Broadly, NDDs are characterized by progressive declines in cognitive, memory, and/or motor functions (12). The most common forms of these diseases are both sporadic and late onset, but specific mutations in the genes encoding various disease-associated proteins are risk factors for NDDs that can result in symptomatic disease earlier in life and in some cases are causative, giving rise to early onset familial forms of these diseases (13). Within this review, we focus principally on the two most common NDDs, Alzheimer’s disease (AD) and Parkinson’s disease (PD), and the misfolding and aggregation of their corresponding disease-associated proteins. These NDDs have unique and overlapping clinical presentations, as well as different pathological features, but they share the progressive formation of amyloid-type fibril aggregates as a defining feature (Fig. 1*B*) (14, 15). Specifically, AD is associated with the formation of fibrils of the microtubule-associated protein Tau (or Tau) and amyloid-β (Aβ) peptides, whereas PD predominantly features fibrils of α-synuclein (α-Syn). The exact causes and locations of the initial protein aggregation in sporadic cases of NDDs are varied and remain ill defined, but there is an ever increasing body of evidence supporting the progressive spread of fibrils that drives further protein aggregation within connected brain regions (16). This process of protein aggregation results in the dysfunction of multiple cellular pathways, the engulfment of organelles, and compromises mitochondria, leading to disrupted protein turnover/localization, metabolism, and a range of cellular malfunctions (17, 18, 19). There are also important associations between protein fibril aggregation and hyperactivation of inflammatory immune responses (20), which likely contributes to neuronal death. While the resulting pathologies are multifaceted and complicated, they eventually lead to loss of neurons and atrophy of specific brain regions, which presages resulting clinical manifestation of disease reflected by impaired cognition.

The links between O-GlcNAc and NDDs began to emerge from genetic experiments aimed at understanding the biological functions of OGT and efforts to identify O-GlcNAc-modified proteins. Knockout of OGT was found to be lethal in embryonic stem cells, and *Cre*-recombinase-based knockout strategies showed that OGT is required for embryogenesis in mice (21). These results demonstrated a clear importance for OGT and/or O-GlcNAc in cell survival and development and pushed the field toward using conditional knockout strategies to explore the underlying biology. Relevant to neurobiology, OGT was deleted in neurons during development using *Cre* expression from the Syn1 promoter (22). Some of the mouse pups survived but had poor locomotor function and died 10 days after birth. Notably, the brains of these mice also had Tau hyperphosphorylation, a marker of Tau aggregation and neurodegeneration in AD. In a similar time frame to these genetic experiments, the first successful antibodies against O-GlcNAc modifications were generated (23, 24). Combined with other reagents like lectins and advances in mass spectrometry, these tools enabled the visualization and identification of O-GlcNAc-modified proteins and led to additional discoveries pointing to a potential role for O-GlcNAc in NDDs. Early experiments using the GlcNAc-binding lectin wheat germ agglutinin revealed that Tau is O-GlcNAc modified at a reasonable stoichiometry, suggesting that it might affect Tau hyperphosphorylation and fibril formation (25). At the same time, mass spectrometry revealed that the crystallin members of the small heat shock proteins were also O-GlcNAc modified (26, 27). The subsequent development of chemical tools, like chemoenzymatic modification (28, 29), coupled with further advances in mass spectrometry demonstrated that α-Syn is also O-GlcNAc modified (30). Anti-O-GlcNAc antibodies were used to show that cells increase O-GlcNAc in response to a variety of stressors, including heat stress, further indicating that the post-translational modification might be playing a role in preventing protein unfolding or aggregation (31, 32). The same antibodies were also used to demonstrate O-GlcNAc levels in brain tissue from AD patients were significantly lower than in age-matched controls (33).

Here, we review how early fundamental findings within the field have been built upon to advance the field from intriguing observations to multiple O-GlcNAc-centered clinical trials for NDDs. Specifically, we will focus on how chemical methods, in particular how small-molecule inhibitors and protein chemistry, contributed to these advances. We direct readers interested in how other more “biology-only” experiments were also indispensable to these efforts to other selected reviews (34, 35, 36).

## Methods for modulating O-GlcNAc in cells and *in vivo*

Genetic methods for manipulating O-GlcNAc levels within cultured cells and *in vivo* have been widely used to gain insight into O-GlcNAc biology. Early studies showed that complete loss of function of either of these genes resulted either in loss of viability during embryogenesis, for OGT (21), and perinatal lethality, for OGA (37). Accordingly, genetic approaches have turned to using more targeted or temporally regulated strategies. Transient transfection of transgenes or shRNA or use of siRNAs have all been exploited. More efficient strategies, including tissue-specific deletion of genes encoding O-GlcNAc-processing enzymes or use of systems enabling inducible knockout, have been pursued, though these later strategies have been more extensively applied to OGT (38, 39, 40, 41, 42). Transgenic mice, in which the OGT gene has been flanked by LoxP sites, enable excision of an essential exon of OGT upon expression of Cre recombinase (21). Most relevant is a study in which OGT was deleted postnatally within forebrain neurons by placing Cre expression under the control of the CamKII promoter. This targeted knockout of OGT gave mice that matured but which started to develop some of the pathological hallmarks associated with neurodegeneration after ∼2 months (43). These included hyperphosphorylated Tau, increased Aβ levels, and higher inflammatory markers, all similar to observations made in AD patient brains. Cre activity can also be controlled by small molecules in various ways, including through fusion to a mutant estrogen receptor that only traffics to the nucleus upon binding of tamoxifen (38). These mice and corresponding mouse fibroblasts are important resources for the community. Unfortunately, neither mice nor cells that have been similarly engineered are available for controlling expression of OGA, though doxycycline-controlled shRNA to OGA has been generated (44). In any case, it is notable that, at least for OGT, the noncatalytic roles of this enzyme have been unambiguously shown to play essential roles in cell physiology (45). The noncatalytic roles of OGA remain less clear, though it has been shown that replacement of OGA with a variant lacking catalytic activity shows the same perinatal lethality as a complete knockout (46), suggesting that loss of catalytic activity contributes to the lethal phenotype. Perhaps most relevant is that loss of OGA in brain, using a brain-specific promoter, resulted in mice that matured but showed a distinct pleiotropic phenotype including metabolic defects along with delayed neurogenesis and brain differentiation that manifested as developmental delay (41). Notably, these mice had dramatically elevated O-GlcNAc within brain, with a fold increase far greater than what is seen when using pharmacological manipulations.

## Pharmacological methods—OGA inhibitors

**Figure 2:**
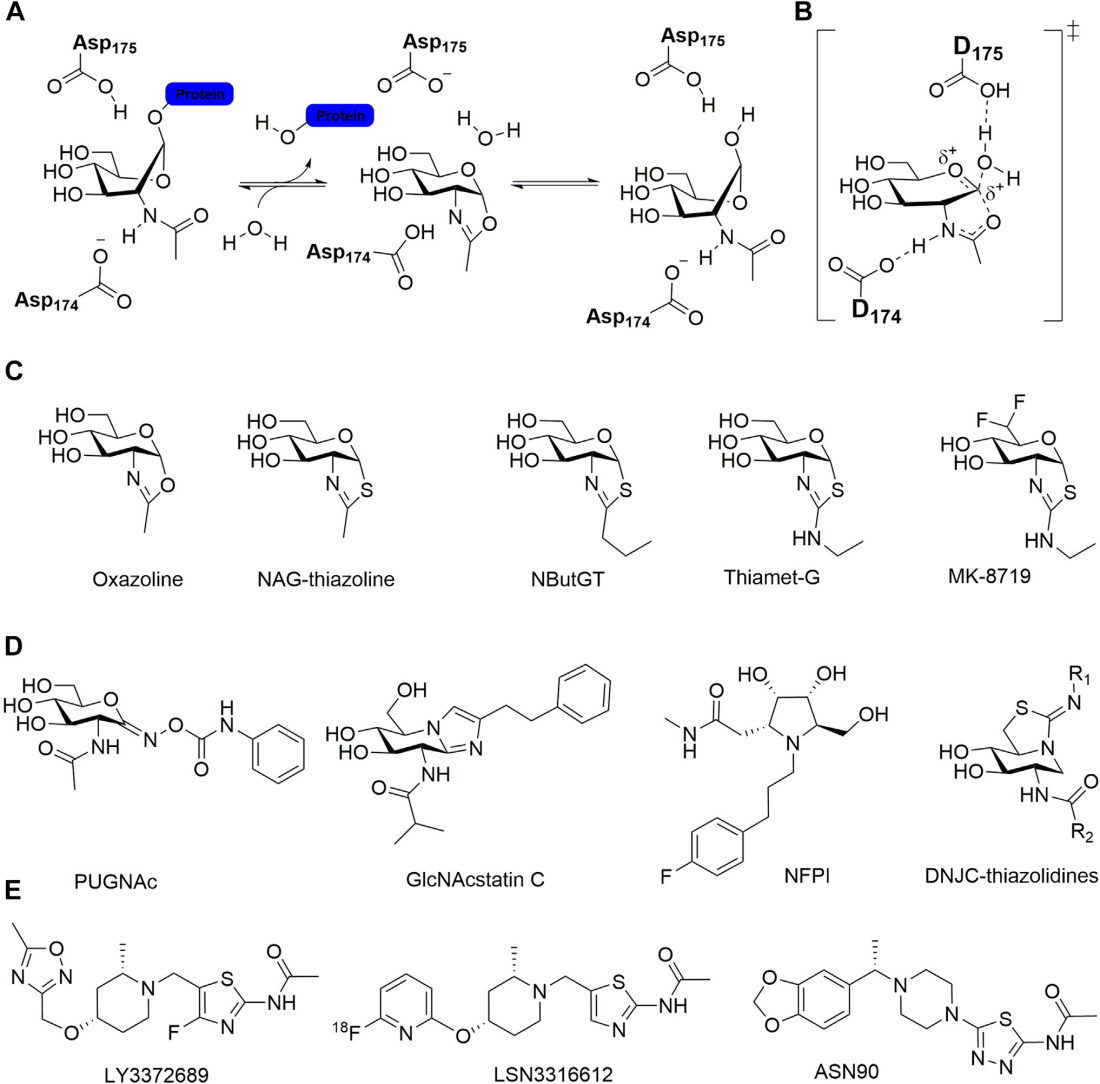
**Selected examples of potent small-molecule inhibitors of O-GlcNAcase (OGA).***A*, OGA uses a two-step catalytic mechanism involving two catalytic aspartate residues acting as general acids/bases. *B*, the proposed transition state structure used by OGA to facilitate substrate hydrolysis. *C*, structure of the oxazoline intermediate present in the reaction pathway used by OGA and bicyclic thiazoline inhibitors inspired by this intermediate, including first-in-class clinical OGA inhibitor MK-8719. *D*, other classes of potent carbohydrate-based OGA inhibitors. *E*, medicinal chemistry–derived inhibitors of OGA that have advanced to the clinic including the PET agent LSN3316612. PET, positron emission tomography.

The creation of selective inhibitors of OGA has contributed to acceleration of research into the potential pharmacological benefits of enhanced O-GlcNAc levels. Extensive knowledge regarding the catalytic mechanisms (Fig. 2, *A* and *B*) and the availability of promiscuous small-molecule inhibitors of glycoside hydrolases led to the early identification of O-(2-acetamido-2-deoxy-d-glucopyranosylidenamino) *N*-phenylcarbamate (PUGNAc, Fig. 2*C*), which was shown to increase O-GlcNAc levels in cell culture (47). PUGNAc was, however, later shown to have a range of adverse off-target effects (48, 49, 50, 51, 52). The cloning (11) and identification of OGA as being a member of glycoside hydrolase family 84 led to elucidation of its catalytic mechanism (53, 54, 55). These findings in turn enabled the creation of mechanism-inspired thiazoline-derived OGA inhibitors such as NButGT (Fig. 2*B*) (53), which derived their selectivity through exploitation of a conserved active-site pocket not found in other functionally related enzymes (56, 57). Other carbohydrate-derived inhibitors followed, including imidazole-based compounds, which exploited the same active site feature to derive selectivity for OGA (58, 59, 60). Strategic modification of NButGT to generate a slightly more basic aminothiazoline derivative that could benefit from a favorable ionic interaction within the enzyme active site led to the extremely tight binding (K_i_ ∼ 2 nM) and highly selective compound Thiamet-G (Fig. 2*B*) (61), which was shown to be a transition state analog (62). For research use, a striking advantage of NButGT and Thiamet-G is their ability to permeate into the central nervous system to increase O-GlcNAc levels in brain (61, 63, 64). The more recent X-ray structures of human OGA (65, 66, 67) have led to new classes of rationally designed carbohydrate-derived inhibitors, including pyrrolidines, with subnanomolar potency (Fig. 2*C*) (68, 69). A more recent development, stimulated by the strikingly favorable benefits of OGA inhibitors in various models of NDD as summarized later, has been industrial interest in creating OGA inhibitors with desirable pharmacokinetic properties. Efforts from the pioneering partnership between Alectos–Merck on OGA inhibitors led to an extensive effort to improve the permeability of Thiamet-G to mitigate potential concerns regarding sustained inhibition of OGA seen when dosing with Thiamet-G (70). This effort resulted in the identification of the first-in-class clinical candidate MK-8719 (Fig. 2*B*), which ultimately went on to phase I clinical trials (71). Following closely on these efforts has been the emergence of OGA inhibitors derived from high-throughput screening that are more amenable to optimization using medicinal chemistry. These include compounds generated by Asceneuron (ASN90, Fig. 2*D*) (72), Eli Lilly (LY3372689, Fig. 2*D*) (73), and Biogen’s undisclosed clinical compound BIIB113, all of which are structurally related and feature a 2-*N*-acetyl-1,3,4-thiadiazole or 2-*N*-acetyl-thiazoline, appended to a central basic piperidine or piperazine unit that is diversified with a pendent heteroaryl group (74).

## Application of OGA inhibitors to transgenic NDD models

The widespread availability of OGA inhibitors, most notably Thiamet-G, which can be obtained from a wide variety of commercial vendors, has enabled exploration of the therapeutic potential of OGA inhibition in various neurodegeneration disease models. The impetus to explore OGA inhibition as a therapeutic strategy for AD stemmed from two independent works (33, 75) that showed human tau was O-GlcNAc modified and that aggregates of tau within AD brain had no associated O-GlcNAc. The earliest proof-of-concept studies were performed in rodent models using Thiamet-G to show acute increases in O-GlcNAc levels in brain led to transient decreases in tau phosphorylation at specific sites (61). This was followed by studies in which Thiamet-G was chronically administered to JNPL3 mice, which have a transgene from which is expressed a mutant (P301L) form of human Tau that is associated with frontal temporal disease with Parkinsonism linked to chromosome 17 (63). OGA inhibition was surprisingly well tolerated and mice were healthy even when treated with high doses of compound. Moreover, JNPL3 mice treated with Thiamet-G showed a dose-dependent increase in O-GlcNAc that correlated with a reduction in the number and extent of tau aggregates, decreased tau pathology, reduced neurodegeneration and neuroinflammation, as well as a corresponding improvement in motor skills. Since then studies using Thiamet-G have been performed in a range of tauopathy mouse models, including TauP301S mice (P301S) (76) and Tg4510 mice (P301L) (44, 77), with similarly protective effects being associated with OGA inhibition. The most notable observations that expand beyond the initial studies have been that short-term treatment, even after a few days, leads reproducibly to improvements in breathing defects in TauP301S mice that cannot be readily accounted for by effects on tau oligomers or aggregates (76, 78, 79). In the same vein, one notable study, focusing on the known AD pathologies that are seen in Down syndrome patients, showed benefits upon treatment of the well-established Down syndrome Ts2Cje mouse model with Thiamet-G (78). Remarkably, twice daily, intranasal delivery of Thiamet-G decreased both Tau and Aβ pathologies, as well as decreasing 3-nitrotyrosine levels in brain, over just 5 days of treatment. The authors suggested that the effects were driven by enhanced autophagy within brain (78). Importantly, studies have also shown that Thiamet-G and MK-8719 treatment following initiation of disease progression, in a therapeutic mode to affect existing disease, also afforded significant protection against neurodegeneration as measured using a range of endpoints including—most strikingly—decreased tau within cerebrospinal fluid (44) and volumetric magnetic resonance imaging of brain volume (80). In all these studies, the observed reductions in Tau aggregation and altered phosphorylation arise, at least in part, from effects of increased O-GlcNAc within the cells in which Tau aggregates since fluorescent Tau-aggregation reporter human embryonic kidney 293 cells show that OGA inhibition reduced pathological tau phosphorylation and aggregation (81).

OGA inhibitors have also been used in a range of other neurodegeneration disease models including various transgenic AD amyloid models. These include mice that express genes containing various mutations associated with familial Alzheimer's disease (FAD) alone or in combination with mutations in Tau. The models examined include 5×FAD mice that express a variant of human amyloid precursor protein (APP) harboring the Swedish (K670N, M671L), Florida (I716V), and London (V717I) FAD mutations along with the human presenilin-1 (PSEN) harboring two FAD mutations (M146L and L286V) (82, 83); bigenic TAPP (tau amyloid precursor protein) mice expressing human tau with the FTDP P301L mutation and human APP with the Swedish FAD mutation (84); as well as 3×Tg mice expressing human APP with the Swedish mutation, human P301L tau, and human PSEN with the M146V mutation (79). In these studies, OGA inhibition resulted in decreased amyloid pathology that was associated with decreased behavioral decline in a number of cognitive and motor skill tests.

More recently, observations that α-Syn is O-GlcNAc modified (30, 85, 86, 87, 88, 89), coupled with elegant *in vitro* studies showing clear effects of O-GlcNAcylation decreasing the aggregation propensity of α-Syn (more details later) (90, 91), have stimulated application of OGA inhibitors in cell and animal models of Parkinon's disease. Within both cultured SK-N-SH cells and primary neurons, decreased uptake of preformed fibrils of synuclein was observed when knocking down OGA by siRNA or through inhibition of OGA with Thiamet-G (92). Moreover, treatment of mice with Thiamet-G in an intracerebroventricular injection model, with delivery of adeno-associated virus driving overexpression of pathogenic aggregation prone A53T α-Syn, resulted in decreased α-Syn pathology, decreased neurodegeneration, and improvements in motor defects (93). These effects were also replicated by genetic downregulation of OGA. A complementary parallel report using ASN-90 in the Line61 transgenic mice that overexpress wildtype human α-Syn also showed beneficial effects associated with OGA inhibition including reduced astrogliosis and pathological phosphorylation of serine129 of α-Syn, which were also accompanied by improvements in locomotion (72).

These widely reproduced protective effects of OGA inhibition in tauopathy models, and more recently in amyloid and synuclein neurodegeneration models, have stimulated interest in the potential for OGA inhibition in other NDDs. Some notable examples include examination of primary cortical neurons used as a model of Huntingtin's disease, where OGA inhibition corrected nuclear pore defects, increased cell viability, and reduced cell death associated with expression of the Huntington's disease–associated Huntington protein variant Htt82Q (94). These effects are consistent with the protective effects of O-GlcNAc in maintaining the nuclear pore (95, 96) and enabling its permeability (97, 98). Studies examining the effects of O-GlcNAc on the TAR-DNA binding protein 43, which is known to undergo pathological hyperphosphorylation and aggregation in frontotemporal lobar degeneration and amyotrophic lateral sclerosis, have been performed using a *Drosophila melanogaster* model. Genetic augmentation of OGT activity ameliorated amyotrophic lateral sclerosis–associated proteinopathy (99). Thus, enhancing O-GlcNAc levels appears to be generally protective in a range of proteinopathies.

These collective studies have provided strong preclinical support for OGA inhibition as a therapeutic strategy to alter disease progression in a range of NDD, with tauopathies and AD in particular being a major focus. First-in-human studies were performed by Merck–Alectos using MK-8719 in an ascending dose study design with both single and multiple repeat dosing, where it was found to be generally well tolerated. The extent of dose-dependent binding of OGA was determined using the positron emission tomography (PET) agent MK-8553. Asceneuron and Lilly have subsequently advanced their structurally distinct compounds ASN-90 (Fig. 2*D*) and LY3372689 through phase I studies, the latter supported by the Lilly OGA PET agent ^18^F-LSN3316612 (Fig. 2*D*) (100). Lilly has since moved their clinical compound into a large phase II trial focused on AD patients early in disease progression with a treatment duration of over 1 year with a projected minimum 80% engagement of OGA by LY3372689. These studies collectively support inhibition of OGA being generally safe and well tolerated within humans.

## Chemical methods to identify and characterize O-GlcNAc on specific NDD-associated proteins

In addition to the cellular mechanisms described previously, chemical and biochemical approaches have also contributed to a more detailed molecular understanding of the effects of O-GlcNAc on particular proteins. Key to these discoveries are several chemical methods that are worth describing in more detail. The first involves the identification of O-GlcNAc modification sites on proteins from brain tissue, which serves as the foundation for most follow-up biochemical investigations.

**Figure 3:**
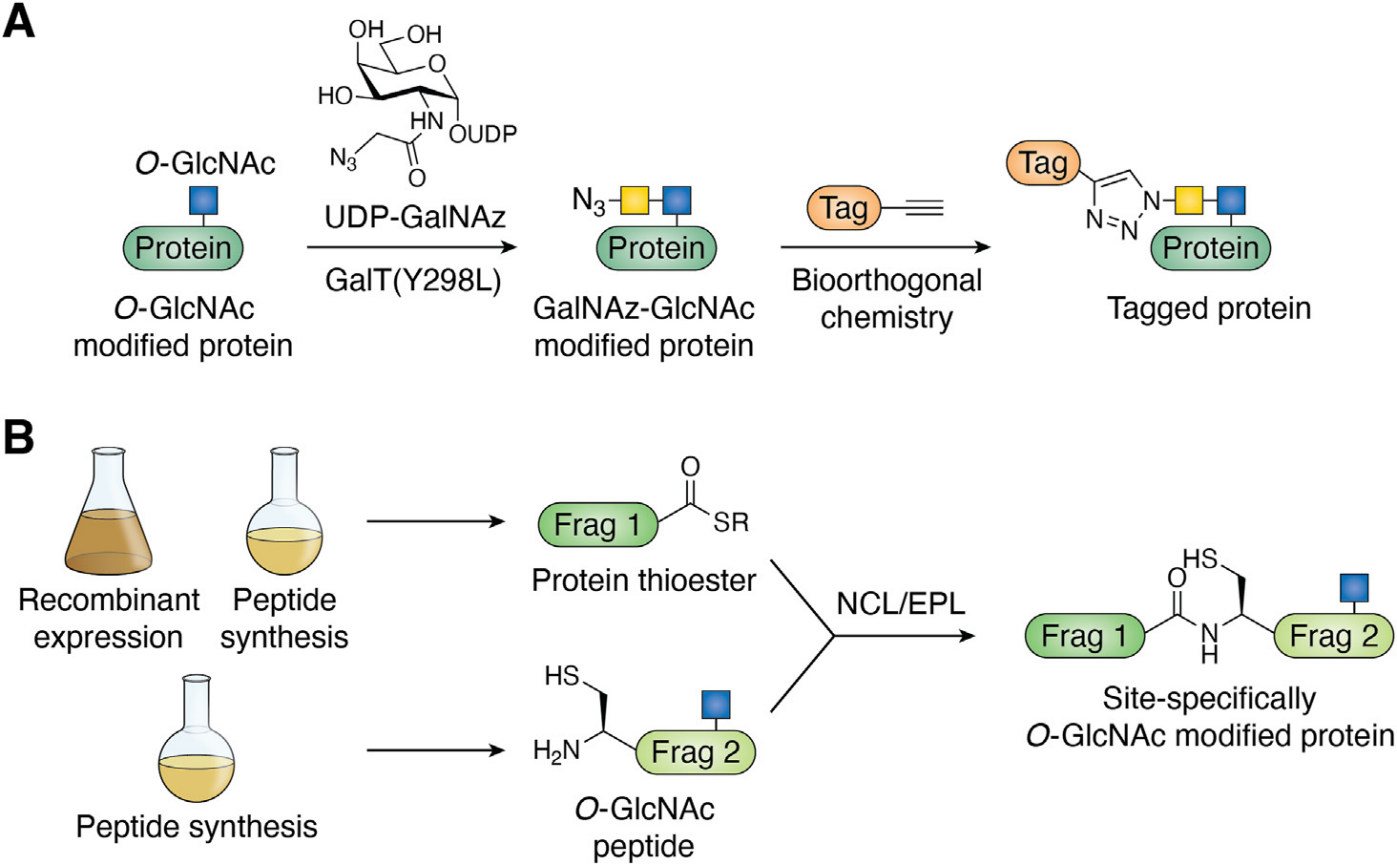
**Chemical methods for studying O-GlcNAc.***A*, chemoenzymatic modification enables the robust detection of endogenous O-GlcNAc modifications through a two-step procedure. *Yellow square*—GalNAz. *B*, site-specifically O-GlcNAcylated proteins can be prepared from synthetic and recombinant fragments using ligation reactions. O-GlcNAc is denoted by a *blue square* and GalNAz by a *yellow square*. EPL, expressed protein ligation; NCL, native chemical ligation.

Detection of the O-GlcNAc modification using commonly used mass analyzers integrated into proteomic platforms results in fragmentation of the glycosidic bond, leading to loss of the O-GlcNAc residue (3, 101). Even with alternative mass spectrometers that leverage fragmentation pathways that spare O-GlcNAc to varying extents (102, 103), identification of O-GlcNAc-modified proteins and sites greatly benefits from the enrichment of modified proteins or peptides from cell or tissue lysates (104). As mentioned previously, the wheat germ agglutinin lectin and antibodies have been used for this type of enrichment; however, they suffer from fairly weak affinity or requirement for underlying peptide epitopes (105), respectively. While progress on this second issue has been recently made by simultaneously using multiple antibodies (106), the precision of chemical methods has greatly contributed to O-GlcNAc enrichment and identification. As mentioned previously, the most robust of chemical techniques that can be applied to endogenous modifications is chemoenzymatic modification (Fig. 3*A*) (28, 29). This technique builds upon the first radioactive GalT experiments that led to the discovery of O-GlcNAc in the first place. Briefly, a mutant of GalT was identified that allows the enzyme to use UDP-*N*-azidoacetyl-galactosamine to modify O-GlcNAc residues, yielding a GalNAz-β1,4-GlcNAc disaccharide. Bioorthogonal chemistry can then be used to tag the azide on GalNAz with biotin for subsequent enrichment of O-GlcNAc-modified proteins using streptavidin. While being the most powerful approach available, there remains some uncertainty regarding the efficiency of the GalT in terms of its ability to stoichiometrically modify all sites of O-GlcNAc.

The second technique is the O-GlcNAc modification of recombinant proteins by coexpressing OGT and a substrate protein in heterologous hosts like *Escherichia coli* (107, 108, 109, 110). For some proteins, this results in O-GlcNAc modification of the substrate protein at one or more sites. This approach can be a straightforward way to generate useful amounts of O-GlcNAc-modified proteins but often suffers from the production of a heterogeneous mixture of protein products that can be challenging to purify. Finally, solid-phase peptide synthesis (SPPS) in combination with protein ligation has been used to overcome this heterogeneity issue for the site-specific installation of protein modifications (111, 112). The iterative and controlled nature of SPPS enables the incorporation of O-GlcNAc at defined positions in the primary sequence. This approach only requires the corresponding serine or threonine building blocks, and these are commercially available or can be prepared using fairly routine chemistry (113). However, standard SPPS is limited to the preparation of peptides of ∼50 amino acids in length. Native chemical ligation was developed to overcome this size limitation by taking advantage of the unique reactivity of peptide thioesters and peptide N-terminal cysteines (114). As shown in Figure 3*B*, these groups will react selectively with each other in aqueous solution with no protecting groups to generate a native amide bond. An extension of native chemical ligation, termed expressed protein ligation, takes advantage of proteins called inteins for the recombinant production of protein thioesters for subsequent ligation reactions (115). Together, these ligation methods allow O-GlcNAc-modified proteins to be built from recombinant and synthetic fragments with complete control over the number and sites of modifications. Though improvements in these methods are making such approaches more widely accessible, these approaches are still most successfully implemented by experts, and in some cases, the location of the site of modification, size of the protein, and problematic refolding of the protein can make this approach impractical. Nevertheless, these synthetic strategies are the gold standard for characterizing the effects of O-GlcNAc on protein function.

## Specific biochemical consequences of O-GlcNAc on tau and α-syn

### Tau

**Figure 4:**
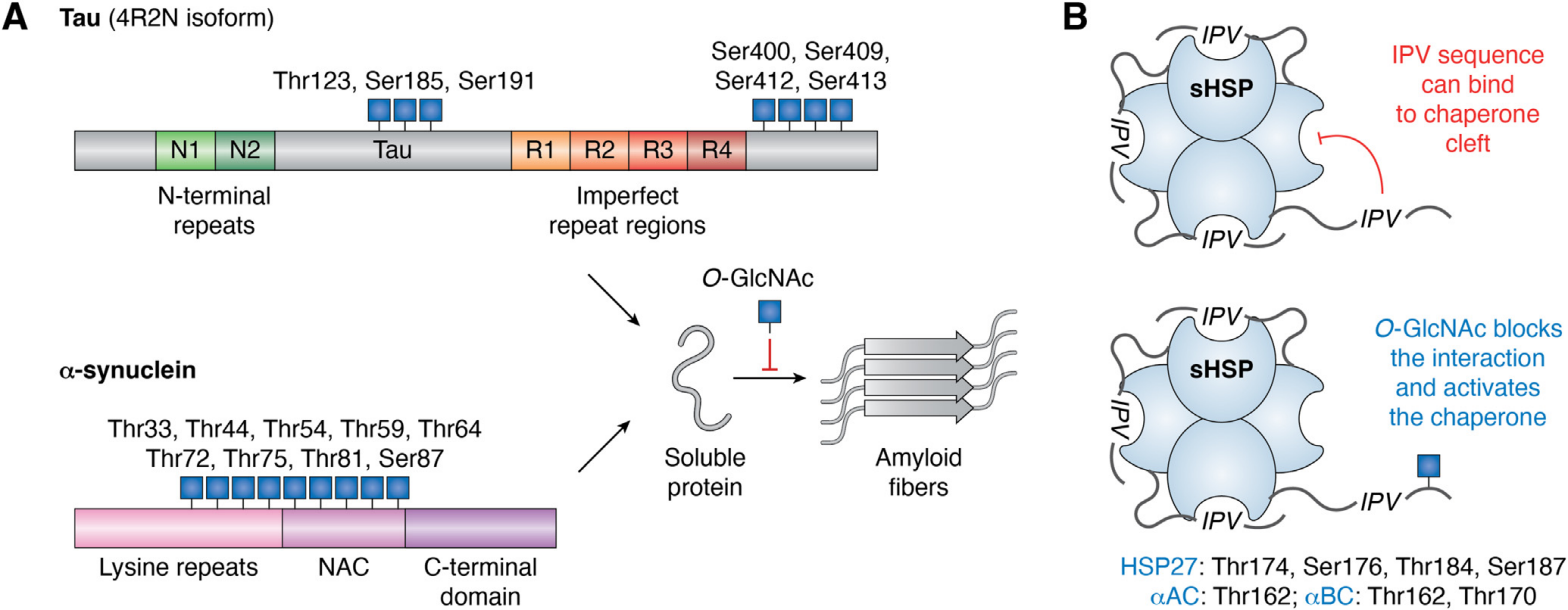
**Consequences of O-GlcNAc on critical proteins in NDDs.***A*, Tau and α-Syn are O-GlcNAc-modified proteins that form amyloid aggregates in AD and PD, respectively. O-GlcNAc modification generally slows the kinetics of fibril formation. The canonical isoform of Tau (4R2N) is shown. *B*, sHSPs are ATP-independent chaperones. O-GlcNAc modification of some sHSPs increases chaperone function by inhibiting the ACD–IPV interaction. O-GlcNAc is denoted by a *blue square*. ACD, α-crystallin domain; AD, Alzheimer’s disease; IPV, Ile–Pro–Val; NDD, neurodegenerative disease; PD, Parkinson’s disease; sHSP, small heat shock protein; α-Syn, α-synuclein.

Tau is a protein involved in the polymerization and stabilization of microtubule structures. Tau in the brain is found as a mixture of six isoforms resulting from alternative splicing. These isoforms differ in the number of N-terminal repeat regions (0N, 1N, or 2N) and the number of imperfect repeat regions (3R or 4R) (Fig. 4*A*) (116). Numbering of the residues in Tau are defined by the longest isoform 2N4R, which contains 441 amino acids. Tau is highly soluble in solution, where it is natively unfolded. However, the protein can oligomerize to form fibrils that make up neurofibrillary tangles (NFTs) found in AD brains (117, 118). Structurally, these fibrils are rich in β-sheets and made up primarily of two amyloid folds, paired helical filaments and straight filaments, with the core of both structures formed by the R3 and R4 repeats. Several lines of evidence link Tau aggregation to the progression of AD and other tauopathies (119). For example, studies have shown that the levels of Tau species in the cerebral spinal fluid or the amounts of NFT in the brain visualized using PET imaging correlate with the onset and progression of a patient’s cognitive decline (120, 121). Several different mouse models of AD have been developed based on the expression of human Tau or aggregation-prone mutants thereof (122).

As mentioned previously, Tau was first identified to be O-GlcNAc modified from bovine brain samples at high stoichiometry (25), but the exact site(s) were only proposed. Later investigations used a combination of OGT coexpression and *in vitro* modification to map Tau O-GlcNAc sites to Thr123, Ser185, Ser191, Ser400, Ser409, Ser412, and Ser413 (107, 123). Thr123 and Ser400 were found to be the major sites of modification using these techniques. Chemoenzymatic labeling was subsequently applied to brain tissue from the JNPL3 AD mouse model, normal rats, and human AD brains (30, 87). This led to the identification of O-GlcNAc at Ser262 and confirmation of modification at Ser400. To test whether O-GlcNAc might directly inhibit the amyloid aggregation of Tau, a recombinant form of Tau spanning residues 244 to 441 was coexpressed with OGT (63). This truncation was used because it spans the amyloid core of Tau, facilitating *in vitro* aggregation reactions, while maintaining the major O-GlcNAc modification site of Ser400. This coexpression led to a mixture of Tau with O-GlcNAc at approximately 50% stoichiometry on Ser400 and one of Ser409, Ser412, or Ser413. The mixture of O-GlcNAc modifications resulted in slower amyloid aggregation than the corresponding completely unmodified Tau protein. In an effort to determine which sites were most important for this result, Ser400 was mutated to alanine, and loss of this O-GlcNAc notably reduced the overall amount of modification and completely reversed the inhibition phenotype. Subsequently, full-length Tau was O-GlcNAc modified using the same recombinant approach, and arduous purification was used to separate the unmodified protein from modified Tau bearing a mixture of 1, 2, or 3 O-GlcNAc modifications (124). Again, O-GlcNAc inhibited Tau fibril formation but did not disrupt the global fold of the protein in solution as measured using FRET. Follow-up NMR characterization of a truncated Tau (residues 353–408) confirmed these results and showed O-GlcNAc only exerted effects on the peptide conformation within the immediate vicinity of the modification site. As mentioned previously, Tau hyperphosphorylation is associated with the formation of NFTs in AD, and treatment with OGA inhibitors can reduce Tau phosphorylation in some models. These results suggested a potential antagonistic crosstalk between O-GlcNAc and phosphorylation at the same or nearby sites. This possibility was directly tested using O-GlcNAc-modified peptides (125). O-GlcNAc modification at Ser400 blocked phosphorylation at the same site as well as Ser396 and Ser404. In the reverse direction, phosphorylation at Ser396, Ser400, or Ser404 prevented the peptide from being O-GlcNAc modified by OGT *in vitro*. Together, these results show that O-GlcNAc can directly inhibit the aggregation of Tau without altering its folded state in solution and that there may be a reverse relationship between O-GlcNAc and Tau hyperphosphorylation. It is likely that these mechanisms contribute to the protective function of OGA inhibitors within preclinical models.

### α-Syn

α-Syn is a short, 140 amino acid, protein that is highly enriched at presynaptic termini with a measured concentration of 50 μM (Fig. 4*A*) (126, 127, 128). Like Tau, α-Syn is natively unstructured in solution; however, in the presence of lipid vesicles, it can form an extended α-helix made up of residues 1 through ∼90 (129). This interaction is driven by imperfect repeats of a lysine-containing sequence KTKEGV that forms an amphipathic helix that interacts with negatively charged lipid head groups. The C terminus of α-Syn is enriched in acidic residues and remains unstructured in both the soluble and membrane-bound conformations. α-Syn was first identified as a 35 amino acid sequence found in aggregates in AD, and this unknown peptide was termed the nonamyloid component (130). The nonamyloid component was eventually matched to residues 61 to 95 of α-Syn, and subsequent biochemical experiments demonstrated that this region drives the fibril aggregation of the protein (131). α-Syn fibrils are the main protein component of aggregate deposits found in several diseases, including PD, multiple system atrophy, and dementia with Lewy bodies (132). Notably, α-Syn fibrils are readily taken up by neurons and can seed the aggregation of endogenous α-Syn, a process that is toxic to the cells and results in the formation of pathogenic deposits that closely resemble those found in diseased brains (19, 133). This process can be reproduced *in vivo*, as α-Syn fibrils injected into mice brains results in spreading of pathology along neuronal connections and development of motor symptoms (134, 135).

Chemoenzymatic modification combined with biotin and proteomics was used in multiple studies to identify up to nine different O-GlcNAc sites from mice brains or human tissue (30, 85, 86, 87, 88, 89). More recently, this same method was combined with PEG mass tags to show that α-Syn is ∼20% O-GlcNAc modified in mouse brain tissue and that this stoichiometry goes up to ∼35% after treatment with an OGA inhibitor (72). Several of these modification sites are located within the core region of the protein responsible for forming amyloids, suggesting that they may inhibit aggregation in a manner similar to that seen for Tau. This possibility was first directly tested using protein ligation focusing on O-GlcNAc modification at Thr72 (90). Specifically, this modified protein was prepared by iterative ligation reactions using one central O-GlcNAc-modified peptide and two flanking recombinant protein fragments. Because α-Syn contains no native cysteine residues required for the ligation reaction, alanine residues were first mutated to cysteine and then transformed back to the native alanine using a desulfurization reaction at the end of the synthesis. This resulted in site-specifically O-GlcNAc-modified α-Syn with no primary sequence mutations. *In vitro* reactions demonstrated that O-GlcNAc at Thr72 slowed the kinetics of amyloid formation. The same approach was separately used to generate α-Syn with O-GlcNAc at Ser87 (136), and site-specific differences on extent of aggregation inhibition were found for the different modification sites. Interestingly, these two sites can also inhibit the cleavage of α-Syn by calpain *in vitro*, a proteolytic event that is associated with α-Syn aggregation in PD (137). To further examine the site-dependent effects of O-GlcNAc on aggregation propensity, four different modified α-Syn proteins with O-GlcNAc at different sites (Thr72, Thr75, Thr81, and S87) were made by protein ligation and characterized in parallel (91). None of the O-GlcNAc modifications altered the unfolded state of α-Syn in solution or prevented the protein from forming an α-helix in the presence of lipid vesicles. As expected from the previous work on Thr72 and Ser87, all the O-GlcNAc modifications inhibited the kinetics of α-Syn aggregation. Specifically, O-GlcNAc at Thr75 or Thr81 was the most inhibitory, whereas modification of Ser87 had the smallest effect. In the same study, α-Syn was synthesized bearing three O-GlcNAc modifications at Thr72, Thr75, and Thr81. This triple modification completely blocked fibril formation and was able to do so even in when combined with the A53T mutant that dramatically accelerates protein aggregation and causes early onset PD. Finally, the *in vitro* inhibition was largely validated as being important, as O-GlcNAc lowered the toxicity of α-Syn toward primary neurons in culture when cells were treated with modified protein that was mixed with unmodified preformed fibrils. Somewhat surprisingly, the exact structure of the sugar residue may play a role in antagonizing oligomerization. Specifically, α-Syn was synthesized with either O-GlcNAc, β-O-glucose, β-O-GalNAc, or α-O-mannose at Thr72. Modification with all these monosaccharide residues inhibited aggregation, but they had different effects, with O-GlcNAc being the most inhibitory overall in a variety of assays (138).

The study of multiple O-GlcNAc sites described previously also found that O-GlcNAc at Ser87 forced α-Syn to form an amyloid aggregate with a different structure compared with unmodified protein as determined by limited proteolysis with proteinase K (91). This result is notable since α-Syn can form different amyloids in different diseases. For example, the α-Syn fibrils isolated from PD patients are quite different structurally from the α-Syn fibrils from multiple system atrophy (139, 140, 141). These and similar observations made studying Tau have led to the hypothesis that specific amyloid structures may drive pathogenesis and direct the course of disease. As mentioned previously, this can be tested through treatment of neurons with fibrils (19, 133) or their injection into mouse brains (135, 142), where they can seed aggregation of endogenous protein and spread to connected brain regions. Recently, this type of experiment was used to characterize the pathogenicity of the α-Syn fibrils formed by O-GlcNAc at Ser87 (143). Strikingly, these fibrils could seed aggregation of unmodified α-Syn *in vitro*, but they showed dramatically lower seeding and pathogenicity when added to neurons in culture or injected into mouse brains. Subsequent cryo-EM analysis confirmed that the fibril structure is quite different from previously characterized aggregates. Overall, these studies using synthetic proteins show that O-GlcNAc may protect from PD and other synucleinopathies in a potentially multifaceted way by both inhibiting the kinetics of aggregation and promoting the formation of fibrils with reduced pathogenicity *in vivo*.

## Other potential mechanisms of neuroprotection

The growing literature provides a remarkably consistent view that pharmacological augmentation of O-GlcNAc levels in cell and animal models alters the underlying progression of various NDDs. In addition, experiments with O-GlcNAc-modified Tau and α-Syn have clearly shown an inhibitory effect on protein aggregation and pathology. Less clear, however, are other potential molecular mechanisms that confer protection in these diverse models. As also noted previously, O-GlcNAc has been found to exert beneficial effects during even short-term treatment of transgenic models that cannot be accounted for by such direct effects on the aggregation propensity of amyloidogenic proteins. These observations have stimulated efforts to identify other cellular processes that are influenced by increased O-GlcNAcylation that could account for some of these more near-term protective effects. Most salient in this regard is seminal work showing that increased O-GlcNAc is a prosurvival pathway that is augmented by a range of stresses including, for example, heat, osmotic stress, oxidative stress, and hypoxia (144). Readers are referred to an excellent review on the general linkages between stress responses and O-GlcNAc (32). In this review, we will focus on cellular processes and pathways most closely linked to NDD-modifying effects pertaining to amyloidogenic proteins.

### Heat shock proteins

Chaperone proteins collectively serve as one of the endogenous mechanisms that have evolved to protect cells from the stresses arising from protein misfolding and aggregation. Heat shock proteins (HSPs) are one class of chaperones that are upregulated in response to a variety of cell stressors that induce protein unfolding or misfolding (145). The expression of several HSPs increases in the brain during aging and is further elevated in neurodegeneration (146), suggesting their upregulation to serve a protective role that ultimately proves futile. There are several families of HSPs, including the ATP-dependent large HSPs like HSP70/90 that can refold proteins, and the small HSPs (sHSPs) that function as “holdases,” which bind hydrophobic patches to block protein aggregation (147). sHSPs function as multimers, ranging from dimers to larger oligomers, and are comprised of three domains: a central α-crystallin domain (ACD) and two flanking unstructured domains (Fig. 4*A*). The ACD is largely responsible for binding of client proteins in a β-sheet cleft, including the amyloid-forming proteins Aβ, Tau, and α-Syn (148, 149, 150). The unstructured domains are important for mediating oligomerization of sHPS and their regulation. Many sHSPs can inhibit fibril formation, and several of these proteins can be found as components of protein aggregates within the brain (151, 152), presumably arising from their coaggregation with amyloid proteins as their chaperone activities are overwhelmed.

Three sHSPs, HSP27, α-crystallin A, and α-crystallin B, have an evolutionary conserved Ile–Pro–Val (IPV) motif within their disordered C-terminal domains. This IPV sequence can bind into the structured cleft of the ACD to modulate the oligomeric state of these sHPs as well as their chaperone activity (150, 153, 154, 155). This property sets up a model where the sHSP is in an off state, in which the IPV is bound within the ACD of the same sHSP, until client proteins build-up to sufficient concentrations where they can compete away the self-associated IPV from the ACD. As noted previously, sHSPs have long been known to be O-GlcNAc modified, and more recent proteomics experiments have localized these sites as being adjacent to the IPV sequence (88, 156, 157). This raised the possibility that O-GlcNAc could antagonize the IPV–ACD interaction and thereby promote sHSP chaperone activity. To test this idea, protein ligation was used to generate site-specifically O-GlcNAc-modified variants of these sHSPs: four different sites on HSP27, one site on α-crystallin A, and two sites on α-crystallin B (158). Biophysical examination using surface plasmon resonance and isothermal calorimetry of these synthetic glycoproteins confirmed that O-GlcNAc proximal to the IPV sequence does indeed inhibit its interaction with the ACD cleft, supporting this hypothesis. Strikingly, all these O-GlcNAc modifications increased the chaperone activity of these sHSPs against the aggregation of Aβ and α-Syn, with the sites closest to the IPV showing the greatest effect. Furthermore, by mixing pure synthetic O-GlcNAc-modified HSP27 with unmodified HSP27 enabled creating a mixture in which O-GlcNAc is present at various substoichiometric levels, allowing the formation of HSP27 oligomers that are mixtures with defined ratios of modified and unmodified monomers (158). Importantly, even at these substoichiometric levels of O-GlcNAc, enhancement of chaperoning by HSP27 was observed. These experiments demonstrate that O-GlcNAc can inhibit amyloid aggregation, not only directly by modification of the aggregation-prone proteins but also through the activation of at least some sHSPs, including HSP27 and α-crystallin B, which are also known to be expressed in neurons. Notably, because even small amounts (200–500:1) of sHSPs can prevent fibril formation, this gain of chaperone function may be a critical feature in the protective role of enhanced O-GlcNAc levels induced by O-GlcNAc in NDDs.

### Autophagy

**Figure 5:**
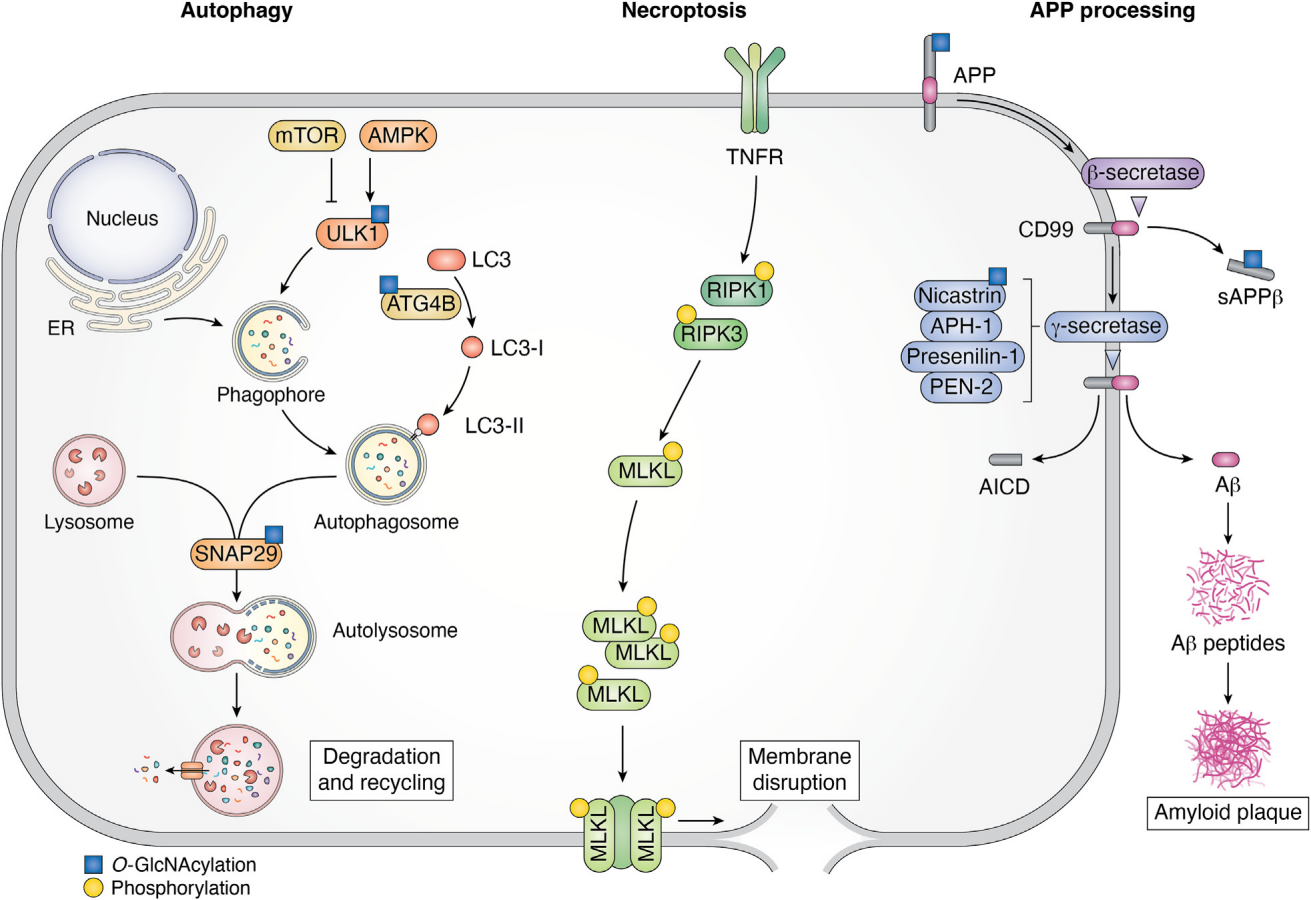
**Roles of O-GlcNAc in autophagy, necroptosis, and APP processing.** Selected components of cellular pathways that have been proposed to be perturbed by increased O-GlcNAcylation that are thought to drive the neuroprotective effects of OGA inhibitors in preclinical neurodegeneration disease models. Proteins within each pathway that are known to be O-GlcNAc modified are marked with a *blue square*, and proteins that are phosphorylated are marked with a *yellow circle*. APP, amyloid precursor protein; OGA, O-GlcNAcase.

Autophagy is a conserved homeostatic cellular pathway that is used to degrade unnecessary intracellular proteins and other cellular components. A dedicated pathway, comprising a diverse and functionally distinctive set of autophagy-related proteins (ATG), coordinates the formation from the endoplasmic reticulum of what is termed the phagophore, which then expands and encloses targeted cellular structures within an autophagosome, which ultimately fuses with lysosomes to generate degradative autolysosomes (Fig. 5) (159). Importantly, autophagy has been shown to degrade amyloidogenic proteins including Tau and α-Syn. Moreover, impaired autophagy has been noted in both synucleinopathy and tauopathy models and patients (160). Augmentation of autophagy in synucleinopathy (161) and tauopathy cell (162) and animal (163, 164) models using a range of pharmacological agents, including various inhibitors of the mammalian target of rapamycin, decreases these pathologies and slows neurodegeneration (165). Accordingly, regulation of autophagy shows potential to alter the course of disease progression in NDDs. Moreover, enhancement of autophagy and the degradation of toxic species may also account for some of the near-term beneficial effects of OGA inhibitors seen in disease models.

The majority of data on the effects of increased O-GlcNAc on autophagy have been obtained using genetic perturbation through either knockdown of OGA or overexpression of OGT. At a high level, the literature presents a somewhat complex picture, driven in part perhaps by the challenges associated with accurately monitoring autophagic flux (166), the disparities between genetic manipulation and pharmacological inhibition of this pathway, as well as variations in cell physiology among the cell and animal models being examined.

Early reports using *Drosophila* as a model showed that increasing O-GlcNAc levels through overexpression of OGT levels led to decreased levels of autophagy-related subcellular features as well as the converse effect upon deletion of OGT (167). This observation, while not addressing autophagic flux, presaged a range of studies in various cell and animal models. Genetic studies in *Caenorhabditis elegans* suggested a complex regulation of autophagy by O-GlcNAc but pointed to O-GlcNAc acting, overall, as a negative regulator of autophagy (168). Further studies indicated that deletion of OGT in *C. elegans* and HeLa cells led to a decrease in autophagic flux (169). Knockdown of OGA or inhibition of OGA using Thiamet-G was also reported as blunting autophagic flux within bladder cancer cells (170) and cardiomyocytes (171). More relevant to NDDs was that altered levels of O-GlcNAc engineered by transfection with OGA and OGT revealed an inverse relationship between O-GlcNAc levels and autophagic flux (172). Furthermore, using Thiamet-G to inhibit OGA and increase O-GlcNAc levels reportedly impaired autophagy, resulting in accumulation of a-synuclein within primary rat cortical neurons (173). Notably, however, this later observation does not manifest in mice treated with OGA inhibitors, which show decreased α-Syn pathology (72, 93).

On the other hand, studies involving knockout of OGA, overexpression of OGT, and OGA inhibitors have in several cases been found to enhance autophagy in a range of models. For example, within liver (174), knockdown or deletion of OGT blocked autophagy, which was also seen in cardiomyocytes (175) and kidney cell (176) models, suggesting that O-GlcNAc is required for autophagy. More relevant for NDDs, knockout of OGA in *C. elegans* (168) and OGA inhibitors used in neuroblastoma SH-SY5Y cells (177), N2a cells (79), primary rat neurons (79) and within the brains of different mouse models (78, 79), all induced autophagy using a wide range of autophagy assays and markers.

The collective data provide tantalizing suggestions that this pathway could mediate some of the protective effects of increased O-GlcNAc levels mediated by OGA inhibitors in models of NDDs. Several studies have proposed specific roles for O-GlcNAc on certain proteins (Fig. 5) including, for example, ULK1 (176), Snap29 (169), and ATG4B (177). Another report has suggested that OGA inhibitors induce autophagy in a mammalian target of rapamycin–independent manner (79). However, while the number of reports slowly increase, the discordant effects that have been observed point to the need for further studies to disentangle the effects of genetic and pharmacological perturbations, perhaps focusing on physiologically translatable transgenic models of NDDs and postmortem patient tissues.

### Necroptosis

Necroptosis is a regulated form of cell death, driven by inflammation, in which the contents of cells are released into the extracellular space. This mode of cellular suicide is driven by the assembly of the necrosome, which forms upon phosphorylation of three essential proteins that are downstream of the death domain receptors: pseudokinase mixed lineage kinase domain-like protein (MLKL), receptor-interacting protein kinase 1 (RIPK1), and RIPK3 (Fig. 5) (178). The phosphorylated necrosome complex migrates to the cell membrane causing it to burst. This pathway of programmed cell death protects against pathogens by destroying host cells and stimulating localized inflammation, but it is also often aberrantly activated in NDDs (179) and is associated with several pathological hallmarks including neuroinflammation and neuronal cell death. Notably, blockade of necroptosis provides benefit in transgenic models of NDDs (179, 180). Moreover, the inflammatory environment induced by necroptosis is known to induce a shift in microglial function from an anti-inflammatory M2 state, in which they normally act as clear protein aggregates (181), to a pro-inflammatory M1 state in which they lose this capacity. Notably, augmentation of O-GlcNAc levels in different cell types, including red blood cells (182), macrophages (183), as well as within brain (83), all reduced necroptosis. Of greatest relevance to NDDs, increased O-GlcNAc induced either genetically or pharmacologically blocked necroptosis in the 5×FAD transgenic model, decreasing overall Aβ burden and amyloid plaques, as well as reducing neuroinflammation and neuronal cell loss, whilst also improving cognition (83). Perhaps most strikingly, OGA haploinsufficiency in these 5×FAD mice resulted in a modest elevation of brain O-GlcNAc that was accompanied by the aforementioned beneficial effects, which suggests that modest levels of pharmacological blockade, ∼50%, could provide therapeutic benefits.

### Altered processing of the APP

Various further reports have suggested alternative pathways that could account for some of the protective effects of increased O-GlcNAc. Without discussing these exhaustively, some studies have focused on the effects of O-GlcNAc on the processing of the APP by the Ɣ-secretase complex (Fig. 5). Some reports have found APP itself is modified though, curiously, all the proposed sites appear to be within the extracellular domain (184, 185). Others have shown that nicastrin is O-GlcNAcylated and proposed that this modification attenuates Ɣ-secretase activity (82). Some studies, using SH-SY5Y and Chinese hamster ovary cells expressing the APP with the Swedish mutation, which accordingly produce high levels of Aβ40 and Aβ42, have found that pharmacological increases of O-GlcNAcylation favors nonamyloidogenic processing of APP (184, 185). Other reports using Thiamet-G have found no effect on APP processing associated with increased O-GlcNAc within primary hippocampal neurons and SK-N-SH cells (79). A more recent report in 5×FAD mice found no effects of OGA inhibition on APP processing and, instead, proposed decreased Aβ burden, and lower numbers of amyloid plaques were due to impaired necroptosis leading to phagocytosis of amyloid oligomers by M2-biased microglia (83). Given the varied results, further studies will likely be necessary to clarify the effects of OGA inhibitors on APP processing.

## Conclusions

As detailed previously, increasing O-GlcNAc has become established as a potentially powerful approach to block the progression of NDDs. Its ability to affect multiple pathways that are common to a variety of NDDs—aggregation, autophagy, cell death, and so on—should encourage the continued development of OGA inhibitors and their preclinical and clinical testing in this class of disease. The pathway(s) that principally contribute to the neuroprotective phenotypes associated with upregulating O-GlcNAc are still unclear, yet we expect that multiple mechanisms are likely contributing to the observed protective effects. An important consideration for these potential drugs is the long-term consequences of OGA inhibition throughout the body, which would chronically elevate O-GlcNAc in tissues beyond the brain. Healthy cells appear to be programmed to maintain overall O-GlcNAc levels within a “normal” range. For example, inhibition of OGT results in increased OGT levels and decreased OGA levels, as the cells seek to return to higher levels of overall O-GlcNAc modification. This is accomplished through O-GlcNAc control of intron retention in the OGT and OGA mRNAs (186, 187). Therefore, it is possible that some healthy tissues may be able to compensate for OGA inhibition. Indeed, as noted previously, extensive preclinical studies have shown OGA inhibition is well tolerated over extended dosing periods. Furthermore, mice with only one functional allele of OGA show normal development and behavior (83). Finally, multiple phase I clinical trials have shown that OGA inhibitors are well tolerated within humans (70, 71, 73). Accordingly, it is hoped that larger ongoing clinical trials of longer duration, which have been supported by preclinical safety studies in multiple preclinical species, will confirm that OGA inhibition is indeed well tolerated by patients. Such findings would support further effort to advance OGA inhibition to treat NDDs, which remain an important unmet medical need.

In addition to the significant efforts around OGA inhibitor development, future work should continue to focus on understanding the cellular and physiological effects of increased O-GlcNAc so as to anticipate potential problems in clinical application of methods to manipulate O-GlcNAc. These efforts should include studying experimental systems ranging from *in vitro* biochemistry to cells and animal models. Such fundamental studies, coupled with research that clarifies the principal protective pathways mediated by increased O-GlcNAc may also enable more targeted therapeutic approaches. Overall, the results from these studies will provide a firm foundation for the appropriate application and evaluation of OGA inhibitors in humans for NDDs and, most likely, other diseases. We believe that the chemical methods described in this review will be indispensable in these efforts.

## Conflict of interest

D. J. V. is a cofounder of and holds equity in the company Alectos Therapeutics. D. J. V. serves as CSO and Chair of the Scientific Advisory Board of Alectos Therapeutics. D. J. V. may receive royalties from SFU for commercialization of technology relating to OGA inhibitors. The authors declare that they have no conflicts of interest with the contents of this article.
